# Hysteroscopy in Postmenopausal Bleeding

**DOI:** 10.4103/0974-1216.71614

**Published:** 2009

**Authors:** Sunita Tandulwadkar, Prashant Deshmukh, Pooja Lodha, Bhavana Agarwal

**Affiliations:** Ruby Hall IVF & Endoscopy Center, Ruby Hall Clinic, Pune, India

**Keywords:** Avoiding hysterectomy, hysteroscopy, hysteroscopy-guided biopsy, postmenopausal bleeding

## Abstract

**Objectives:**

1) To study the etiology of postmenopausal bleeding (PMB). 2) To study the significance of hysteroscopy in evaluation of the etiopathogenic factors. 3) Correlating the diagnosis after transvaginal sonography (TVS), hysteroscopy and histopathologic diagnosis. 4) Feasibility of conservative management with hysteroscopy in PMB.

**Design:**

Prospective study from January 2008 to June 2009.

**Setting:**

Department of Obstetrics and Gynecology of Ruby Hall Clinic, Pune.

**Patients:**

Sixty postmenopausal women with complaint of bleeding per vaginum.

**Interventions:**

Clinical and sonographic evaluation, followed by diagnostic and/or therapeutic hysteroscopy and guided biopsy. Hysteroscopic images were analyzed and compared with histopathologic results.

**Measurements and Main Results:**

On hysteroscopy, endometrium was classified as suggestive of normal, atrophic, endometrial hyperplasia or endometrial carcinoma. Histopathologic diagnosis is taken as a gold standard to determine the efficacy of hysteroscopy in diagnosing endometrial pathologies. The sensitivity and specificity of hysteroscopy in diagnosing endometrial pathologies was assessed.

**Conclusions:**

In women with PMB, hysteroscopy is the basic tool that allows precise diagnosis of various endouterine pathologies. The average sensitivity of hysteroscopy was 97% in our study and the specificity was 98.66%. Hence, we can conclude that it is highly accurate for evaluating endometrial pathologies. For obvious benign lesions, it also provides treatment in the same sitting, therefore avoiding an extensive, morbid, and expensive procedure like hysterectomy.

## INTRODUCTION

A woman is considered menopausal, after cessation of menstruation for 1 year. The average age of menopause in Asian women is 46 years.[1] With increasing life expectancy, a healthy 50-year-old woman today spends as much as 40% of her life in postmenopausal state. During this prolonged period, women are vulnerable to various conditions, of which one of prime importance and sincere concern is postmenopausal bleeding (PMB).[2]

Atrophic endometritis, endometrial hyperplasia and endometrial cancer are the leading causes of PMB.[3]

The goal of evaluation of PMB is to achieve the diagnosis with greatest accuracy, the least risk and expense for the patient. With the advent of hysteroscopy in the last two decades, focus has shifted from endometrial biopsy to hysteroscopic-guided biopsy as a “gold standard” diagnostic tool in the evaluation of PMB.[4]

### Aims and objectives

1. To study the etiology of PMB
2. To study the significance of hysteroscopy in evaluation of the etiopathogenic factors
3. To study the selection of various investigations available and their impact on diagnosis of etiology of PMB
4. Correlating the diagnosis after transvaginal sonography (TVS), hysteroscopy and histopathologic diagnosis
5. Feasibility of conservative management in PMB

## PATIENTS

This was a prospective study of 60 postmenopausal women (at least 1 year of amenorrhea) attending the Gynecology Outpatient Department of Ruby Hall Clinic with the complaint of per vaginal bleeding. This study was carried out over 18 months from January 2008 to June 2009.

### Exclusion criteria

1. Women taking hormonal replacement therapy
2. Obvious cause of bleeding from cervix and vagina
3. K/c/o bleeding dyscrasias
4. Anticoagulant therapy
5. Surgical menopause
6. TVS showing adnexal pathology

For each patient, detailed history was taken, which includes general medical history, menstrual and obstetric history, duration since menopause, severity and duration of PMB, history of gynecologic operations, drug intake and associated symptoms. A thorough general and systemic examination was done, along with abdominal, vaginal and rectal examinations.

Endometrial thickness (ET) was measured in the longitudinal plane on TVS. The adnexal region was also covered in the ultrasonic examination to exclude extrauterine pelvic masses.

Clinical and sonographic evaluation was followed by diagnostic and/or theraupetic hysteroscopy with Office Hysteroscope (Versascope of Johnson and Johnson - Mumbai, India). In each case, hysteroscopy with visualization of the uterine cavity was performed and hysteroscopic-guided biopsy was done. Sometimes, cervical stenosis poses significant difficulty while performing hysteroscopy in postmenopausal women. Paracervical block with 2% xylocaine was used when difficulty was encountered at the level of internal OS.

Records of hysteroscopy finding are tabulated below. Endometrial biopsy of a suspected lesion was taken in all cases. On hysteroscopy, endometrium was classified to be suggestive of:

1. Normal
2. Atrophic
3. Endometrial hyperplasia
4. Endometrial carcinoma – obvious intrauterine growth with necrotic tissue was seen.

Histopathologic diagnosis is taken as a gold standard to determine the efficacy of hysteroscopy in diagnosing endometrial pathologies. In cases of obvious benign lesions like polyp and submucous fibroid, the patient was treated in the same sitting with versapoint.

Thus, sensitivity and the specificity of hysteroscopy in diagnosing various endometrial pathologies were assessed.

## RESULTS AND DISCUSSION

Even a single episode of postmenopausal vaginal bleeding needs a meticulous evaluation. It can be the sole manifestation of the underlying endometrial cancer, which is most probably at a stage when it can be cured completely.

The uterine causes of PMB and the percentage of patients who seek treatment for these conditions are presented in Table 1.

**Table 1:** Causes of postmenopausal uterine bleeding

Causes of bleeding	Percentage	Our study (%)
Atrophic endometrium	60–80	66.66
Exogenous estrogens	15–25	0.00
Endometrial cancer	10	13.3
Endometrial polyps	2–12	11.6
Endometrial hyperplasia	5–10	6.66
Others (cervical cancer, urethral caruncle, trauma, etc.)	5–10	Excluded

Reports in the literature indicate that curettage alone with endometrial biopsy techniques carry false negative rates between 2 and 6% as curettage is a blind procedure and in approximately 60% of curettage procedures, only half of the uterine cavity is curetted.[5] TVS carries a false negative rate of 3%. The fact that curettage operations have limitations in the diagnosis of endometrial polyp and other pathologic conditions indicates the need for a minimally invasive and the most accurate method like hysteroscopy for the evaluation of the uterine cavity in women with PMB. Also, TVS is unreliable as there is subendometrial edema, which makes it difficult to get an accurate measurement of the true ET.

In our study, we have evaluated all cases of PMB with hysteroscopy with versascope and guided biopsy.

In recent years, interest has been focused on hysteroscopy as a potential minimally invasive technique for use in the diagnostic workup of women with PMB, as a first line of investigation.[6–11]

In our study, women in their 50s formed 56.6% among the subjects with PMB [Table 2]. Five out of 15 (i.e. 33.3%) women of more than 55 years of age were subsequently found to have been suffering from endometrial carcinoma [Table 3]. But none of the postmenopausal patients up to 49 years of age had endometrial carcinoma [Table 3]. Though 50% of the women were overweight, five obese women out of eight (62.5%) were subsequently diagnosed to have endometrial carcinoma [Table 4]. Also, 56.6% of the patients were primiparas, whereas 6 out of 18 (33.3%) nulliparas were diagnosed to have endometrial carcinoma [Table 5]. Risk factors for endometrial cancer like obesity (62.25%), diabetes mellitus (50%), hypertension (25%) were all significantly associated with the occurrence of endometrial carcinoma in our study [Tables 5 and 6].

**Table 2:** Distribution of cases according to the age at menopause

Age of attaining menopause (years)	No. of women with PMB
<45	3 (5)
45–49	8 (13.3)
50–55	34 (56.6)
>55	15 (25)

Figures in parenthesis are in percentage

**Table 3:** Correlation between age of attaining menopause and carcinoma of endometrium

Age of attaining menopause (years)	No. of women with PMB	No. of cases detected with carcinoma of endometrium on histopathology report (HPR)
<45	3	0
45–49	8	0
50–55	34	3 (8.82)
>55	15	5 (33.3)

Figures in parenthesis are in percentage

**Table 4:** Distribution of the cases according to the body mass index

BMI	No. of women with PMB	Percentage
<18.5 (Underweight)	4	6.6
18.5–24.9 (Healthy weight)	18	30
25–29.9 (Overweight)	30	50
>30 (Obese)	8	13.3

**Table 5:** Distribution of the cases according to the parity

Parity	No. of women with PMB	Percentage
Nullipara	18	30
Primipara	34	56.6
Multipara	8	13.3

**Table 6:** Correlation between co-morbid conditions and carcinoma of endometrium

Co-morbid conditions	No. of women with PMB	No. of women with carcinoma of endometrium
Diabetes mellitus	12 (20)	4 (50)
Hypertension	8 (13.33)	2 (25)
Hypothyroidism	3 (5)	0 (0)
BMI > 30 (obese)	8 (13.33)	5 (62.5)

**Figure 1:**
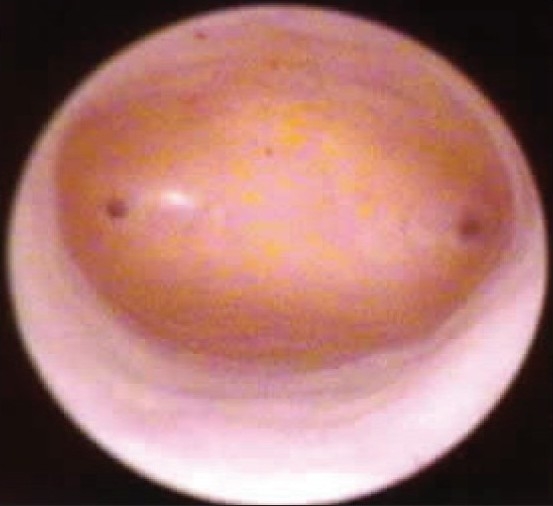
Atrophic Endometrium

Exactly 58.3% of the patients with PMB had a thin endometrium (<5 mm), indicating atrophic endometrium [Figure 1] as the commonest cause[12,13] [Tables 7 and 8]. Out of the seven women with ET > 12 mm, four (57.14%) were diagnosed to have endometrial carcinoma on histopathologic evaluation. All these observations were comparable to the most of the international studies.[14]

The incidence of endometrial carcinoma (13.33%) is comparable to that in a previous study[3] by Pacheco *et al*, in which incidence of endometrial cancer in patients with PMB was 10–14%.

**Table 7:** Correlation between ET on TVS and PMB

ET TVS (mm)	No. of women with PMB	Percentage
<5	35	58.3
5–12	18	30
>12	7	11.6

**Table 8:** Incidence of various suspicious endometrial pathologies on TVS and hysteroscopy causing PMB

Diagnosis	TVS	Hysteroscopy	HPR
Atrophic endometrium	35 (58.33)	39 (65)	40 (66.66)
Endometrial hyperplasia	3 (5)	4 (66.67)	4 (6.66)
Endometrial polyp	5 (8.3)	7 (11.66)	7 (11.6)
Submucous fibroid	1 (1.66)	1 (1.66)	1 (1.6)
Carcinoma of endometrium	4 (6.66)	7 (11.66)	8 (13.3)

Figures in parentheses are in percentage

**Figure 2:**
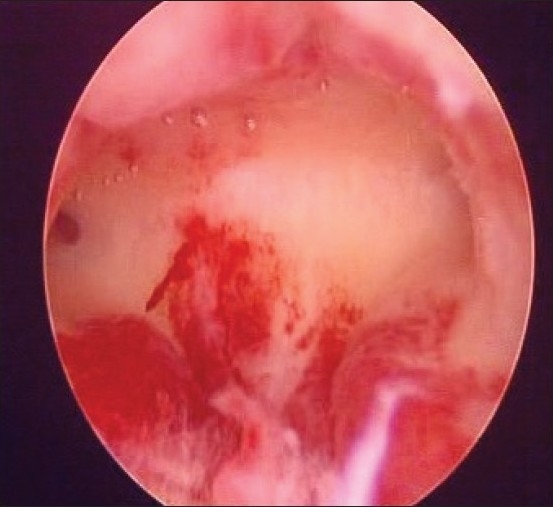
Endometrial Hyperplasia

In our study, endometrial hyperplasia [Figure 2] was found in 6.66% of the patients and endometrial atrophy in 66.6%. Studies conducted in India and other countries of Southeast Asia[12,13] yield similar figures for atrophic endometrium as a cause of PMB. But in the western world, atrophic endometrium is seen in less than half of the patients with PMB, and instead, estrogen replacement therapy accounts for a significant number of patients of PMB.

In our study, the sensitivity of hysteroscopy in diagnosing endometrial hyperplasia and endometrial cancer was 93.75% [Table 9], in accordance with a value of 94.4% obtained in a study by Ribero *et al*, in November 2007.[4,15–18]

**Table 9:** Sensitivity and specificity of TVS and hysteroscopy for diagnosing endometrial pathologies casing PMB

Diagnosis	TVS		Hysteroscopy	
	Sensitivity (%)	Specificity (%)	Sensitivity (%)	Specificity (%)
Atrophic endometrium	87.5	80	97.5	95.23
Endometrial hyperplasia	75	98.2	100	100
Endometrial polyp	71.4	96.36	100	100
Submucous fibroid	100	100	100	100
Carcinoma of endometrium	50	92.8	87.5	98.1

**Figure 3:**
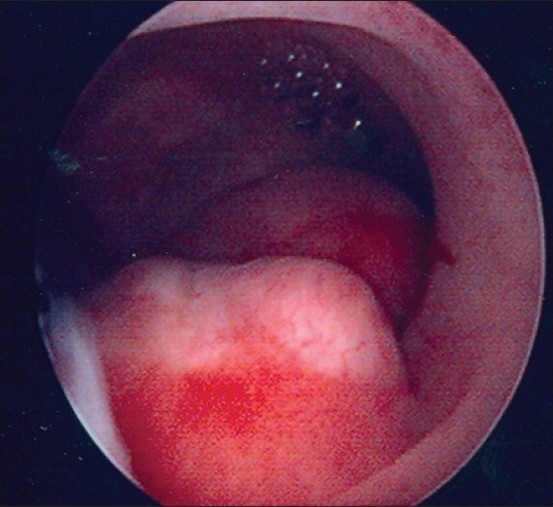
Multiple Endometrial Polyps

It is apparent that hysteroscopy is much more sensitive than TVS in the detection of focal endometrial pathologies such as endometrial polyp [Figure 3] (97 and 76.7%, respectively). Rather, the technical improvements have made hysteroscopy most suitable for office use. Also the specificity of hysteroscopy is more than TVS in diagnosing various endometrial conditions (98.5 and 93.3%, respectively) [Table 9].

**Figure 4:**
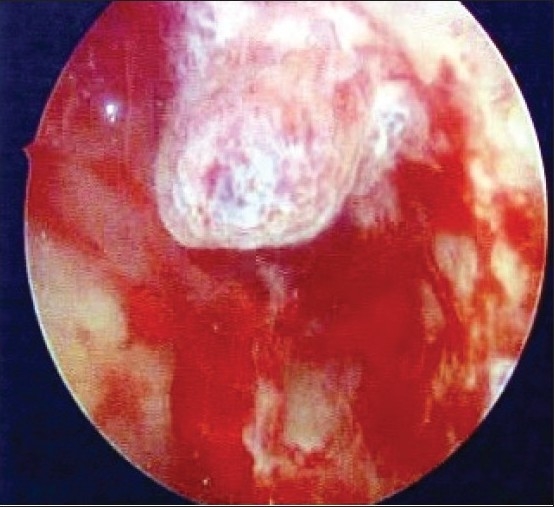
Malignant Endometrial growth

**Figure 5:**
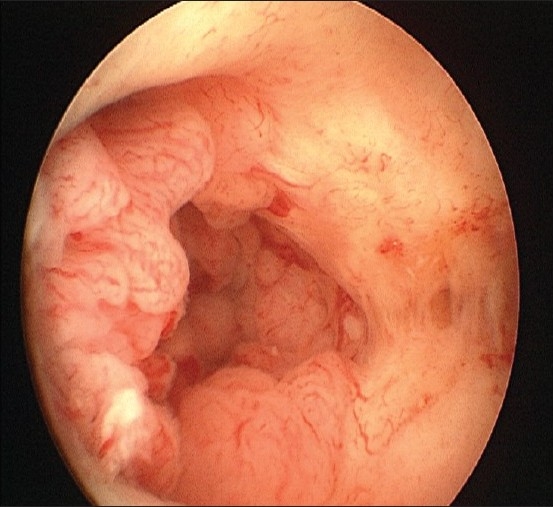
Carcinoma Endometrium

However, there have been cases that deserve attention. Dorum *et al*,[19] reported two such cases of endometrial cancer [Figures 4–5] with ET of <4 mm in their series of 100 women with PMB. Philip *et al*,[20] reported in their study including 85 Jamaican women that half of the patients with endometrial carcinoma had an ET of 3–4 mm. These articles at the same time, discuss the probable reason for this disparity. An occasional patient with repeated episodes of heavy bleeding might have shed her endometrium and hence might be showing a thin endometrium and such cases are picked up on hysteroscopy. This analysis illustrates that endometrial cancers will occasionally be missed if transvaginal ultrasonographic measurement of ET is used as a sole mode of investigation of PMB.

## CONCLUSIONS

1. Women between 50 and 55 years formed the majority (56.6%) of the patients with PMB [Table 2]. But the incidence of carcinoma was highest in those above 55 years of age (i.e. 33.3%) [Table 3]. But none of the postmenopausal patients up to 49 years of age had endometrial carcinoma.
2. Factors such as obesity (62.25%), diabetes mellitus (50%), nulliparity (33.3%) and hypertension (25%) were significantly associated with the occurrence of endometrial carcinoma.
3. Atrophic endometritis was the most common cause of postmenopausal bleeding (66.67%), followed by endometrial carcinoma (13. 6%) and endometrial polyp (11.6%).
4. After correlating clinical diagnosis and diagnosis after investigations (TVS and hysteroscopy), hysteroscopy was found to be the most sensitive (97% vs. 76% of TVS) and specific (98.66%) method for diagnosing endometrial pathologies, considering histopathology to be the gold standard for diagnosis.
5. Hysteroscopy can be considered as the simple, safe, effective and first-line gold standard method for the evaluation of the patients with PMB.
6. In elderly patients who are at high risk for any invasive procedure like hysterectomy, hysteroscopy is effective in reducing the number of hospital visits, admissions and total costs.

Though a larger study with a bigger sample size is definitely recommended, from our study it can be definitely concluded that hysteroscopy should be considered as a first-line modality in the management of the patient with PMB.
